# Complete genome sequences of four *Ralstonia solanacearum* phylotype IIB isolated from Musaceae plants in the Philippines

**DOI:** 10.1128/mra.00915-26

**Published:** 2026-09-14

**Authors:** Kaye Ann L. Alerta, Asuncion K. Raymundo, Walter A. Laviña, Benny M. Corcolon, Dennice G. Catambacan, Andrew D. Montecillo

**Affiliations:** 1 Graduate School, University of the Philippines Los Baños 54729, Los Baños, Laguna, Philippines; 2 Microbiology Division, Institute of Biological Sciences, University of the Philippines Los Baños 54729, Los Baños, Laguna, Philippines; 3 National Academy of Science and Technology Philippines, Bicutan, Taguig, Philippines; 4 Research Information and Compliance Division, TADECO, Panabo City, Philippines; Indiana University Bloomington, Bloomington, Indiana, USA

**Keywords:** *Ralstonia solanacearum*, *Ralstonia solanacearum* phylotype IIB, Moko, Bugtok, banana, complete genome sequence, whole-genome sequence, long-read sequencing

## Abstract

We report complete genome sequences of four *Ralstonia solanacearum* strains from Philippine Musaceae plants. Genomes spanned 5.5–5.6 Mb (66.5% GC) and comprised a chromosome and megaplasmid; three strains additionally carried a small plasmid (146–190 kb). All four isolates were assigned to phylotype IIB-3.

## ANNOUNCEMENT

*Ralstonia solanacearum*, one of the world’s most destructive bacterial plant pathogens, infects over 450 plant species across 54 botanical families (1–3). Here, we report the complete genome sequences of four Philippine banana strains causing Moko and Bugtok diseases: historical isolates Mo-D5 and Bu-D11, collected in the 1990s from Panabo City, Davao del Norte, and Bago Oshiro, Davao City (4); and contemporary isolates Mo-D26 and Bu-D26, collected in early 2026 from the pseudostem of a Cavendish cultivar in Braulio E. Dujali (7° 25′ 4.375″ N, 125° 37′ 14.116″ E) and Cardava fruit in Carmen, Davao del Norte (7° 25′ 4.375″ N, 125° 38′ 57.814″ E), respectively. Contemporary strains were isolated following Vaughan (5), with modifications, and typical *Ralstonia* colonies were purified on Kelman medium supplemented with 1% triphenyl tetrazolium chloride (6) and casamino acid-peptone-glucose (CPG) medium at 30°C for 48–72 h.

**Fig 1:**
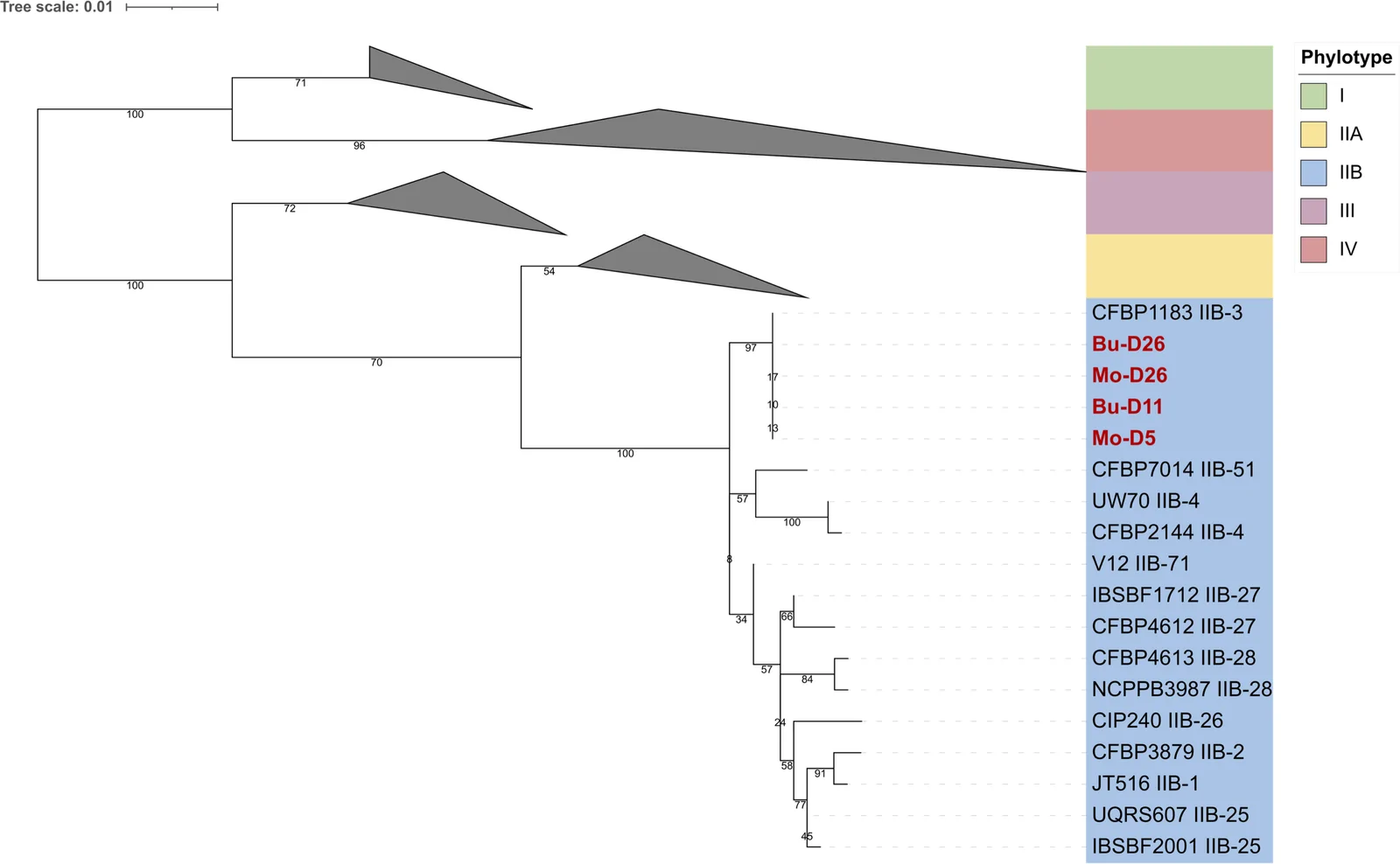
Maximum likelihood phylogenetic tree based on endoglucanase (*egl*) gene sequences of isolates and reference strains. The tree was inferred using RAxML-NG v1.2.2 under the TIM1 + I + G substitution model with 1,000 bootstrap replicates. Branch lengths represent nucleotide substitutions per site (tree scale = 0.01), and bootstrap support values are indicated at the nodes. The four Philippine banana isolates (highlighted in red) clustered together within the phylotype IIB-3 clade with 97% bootstrap support. The tree was visualized using iTOL v7.6.

Default parameters were used in subsequent sequence analysis tools unless otherwise specified. All strains were identified to the genus level by 16S rRNA sequencing (7), with BLASTn against the NCBI 16S rRNA database (8, 9) identifying *R. solanacearum* strain K60-1 (NR_044040.1) as the closest match (98%–99% sequence similarity). Strains were cultured in CPG medium (48 h, 30°C) and submitted to Plasmidsaurus (Singapore) for genomic DNA extraction and hybrid (ONT-Illumina) whole-genome sequencing. Genomic DNA was extracted from each strain with the Zymo Quick-DNA Fungal/Bacteria Miniprep Kit (Zymo Research) according to the manufacturer's instructions. The same genomic DNA extracts were used for both long- and short-read library preparation and sequencing. Short-read libraries were prepared with the Illumina DNA Prep Kit and sequenced on an Illumina NovaSeq X Plus (2 × 150 bp) and quality-filtered with fastp v0.24.0 (10). Long-read libraries were prepared with Rapid Barcoding Kit 96 v14 (SQK-RBK114.96), sequenced on PromethION P24 (R10.4.1) with basecalling by Dorado v7.4.12 (SUP mode), and quality-filtered with Chopper v0.9.2 (11) (-l 1000, -q 10). Sequencing yield, quality, and assembly metrics for each strain are summarized in Table 1. Assemblies were generated with Autocycler v0.4.0 (12), using Canu v2.3 (13), Flye v2.9.5 (14), Miniasm v0.3 (15), NECAT v0.0.1 (16), NextDenovo v2.5.2 (17), and Raven v1.8.3 (18), then polished with Medaka v2.0.1. Polypolish v0.6.1 (19) and Pypolca v0.4.0 (20, 21) were used to polish the assembly with short reads. Assembly quality was assessed with QUAST v5.3.0 (22), SeqKit v2.10.0 (23), Samtools v1.13 (24), and BUSCO (25). Final assemblies comprised two to three closed-circular contigs (5.5–5.6 Mb, 66.5% GC, N_50_ 3.1–3.4 Mb) with 100% complete BUSCOs, each resolving into a chromosome and a megaplasmid; Mo-D5, Mo-D26, and Bu-D26 additionally carried another plasmid (146–190 kb) absent from Bu-D11. NCBI PGAP v6.10 (26) predicted 4,809–4,864 protein-coding genes, 55 tRNAs, and 9 rRNA genes per genome. Mash v2.3 (27) showed 99.98% identity to *R. solanacearum* strain CIP417 (GCF_000825825.1), and ANI >99% (28) with dDDH >70% (29) confirmed all isolates as phylotype II. Phylogenetic analysis based on the endoglucanase (*egl*) gene clustered all four isolates with the phylotype IIB-3 reference strain CFBP1183 (Fig. 1) (30–33). Ralsto T3E (34) identified 57–60 predicted type III effector genes per genome: 59 in Mo-D5, 58 in Bu-D11, and 62 in both Mo-D26 and Bu-D26.

**TABLE 1:** Sequencing yield, quality, and general genome assembly features of four *R. solanacearum* phylotype IIB strains^*a*^

Metrics/features	Mo-D5	Bu-D11	Mo-D26	Bu-D26
Sequencing				
Raw short reads (in millions)	21.95	13.91	26.99	46.73
Filtered short reads (in millions)	21.20	13.75	25.50	44.58
Short-read quality	Q30, 93.4%	Q30, 95.9%	Q40, ≥90%	Q40, ≥90%
Raw long reads	72,108	31,620	22,394	94,750
Long reads N_50_ (kbp)	15.8	20.7	16.0	13.5
Filtered long reads (>1 kb, Q ≥ 10)	62,737	28,248	20,801	62,854
Long reads ≥Q20 (%)	>80	>80	>80	>80
Mean long-read length (kb)	9.2	11.4	6.6	8.4
Long-read genome coverage (×)	105	58	36	94
Short-read genome coverage (×)	593.5	375	729.6	1,263.3
Assembly				
Total length (bp)	5,586,216	5,584,366	5,590,232	5,634,719
No. of contigs	3	2	3	3
Chromosome (bp)	3,111,491	3,257,901	3,383,977	3,384,004
Megaplasmid (bp)	2,328,338	2,326,465	2,059,868	2,060,878
Other plasmid (bp)	146,387	n.d.	146,387	189,837
G + C content (%)	66.5	66.5	66.5	66.5
No. of predicted protein-coding genes	4,809	4,815	4,810	4,864
No. of predicted hypothetical proteins	123	115	124	127
No. of predicted type III effector genes	59	58	62	62

^*a*^ n.d., not detected.

## Data Availability

The complete genome sequences of four *Ralstonia solanacearum* banana strains, Mo-D5, Bu-D11, Mo-D26, and Bu-D26, have been deposited in GenBank under accession numbers GCA_058934505.1, GCA_058934665.1, GCA_058934555.1, and GCA_058934535.1, respectively. The BioProject accession number is PRJNA1480403, and the BioSample accession numbers are SAMN61027274, SAMN61028615, SAMN61028616, and SAMN61028617 for Mo-D5, Bu-D11, Mo-D26, and Bu-D26, respectively. The raw sequencing data for both long reads and short reads utilized in the assembly have been deposited in the NCBI Sequence Read Archive under accession numbers SRR39418895 and SRR39418896 for Mo-D5, SRR39418893 and SRR39418894 for Bu-D11, SRR39418890 and SRR39418891 for Mo-D26, and SRR39418892 and SRR39418889 for Bu-D26. The predicted type III effector proteins identified in the four isolates are available at Zenodo (https://doi.org/10.5281/zenodo.21995664).
